# Trends in health behaviors and mental health among Korean adolescents in Korea over 5 years, 2017–2021: focusing on the comparisons before and during the COVID-19 pandemic

**DOI:** 10.3389/fpubh.2023.1139110

**Published:** 2023-04-17

**Authors:** Mi-Sun Lee, Dong Jun Kim, Hooyeon Lee

**Affiliations:** ^1^Department of Preventive Medicine, College of Medicine, The Catholic University of Korea, Seoul, Republic of Korea; ^2^Department of Public Health, Graduate School, The Catholic University of Korea, Seoul, Republic of Korea

**Keywords:** health behavior, mental health, adolescent, trend, coronavirus disease 2019

## Abstract

**Objectives:**

We investigated recent trends in health behaviors and mental health conditions among Korean adolescents from 2017 to 2021 and compared the changes before and during the coronavirus disease 2019 (COVID-19) pandemic.

**Methods:**

Data analysis was conducted on 289,415 adolescents participating in the Korea Youth Risk Behavior Web-Based Survey, an annual cross-sectional study from 2017 to 2021. All analysis was conducted using sex stratification, and the annual percentage change (APC) was calculated.

**Results:**

Alcohol consumption and smoking decreased in the first year of the COVID-19 pandemic compared with before, except for girls from the low-income level. The prevalence of inadequate physical activity for both boys and girls increased in 2020 compared with the pre-COVID-19 period and decreased again by 2021. The prevalence of obesity in both sexes increased regardless of the period (boys, APC = 8.2%, 95% confidence intervals (CI), 6.4–10.1; girls, APC = 3.3%, 95% CI, 1.8–4.8). The prevalence of stress, depression, and suicidal ideation, plans, and attempts for both sexes decreased in 2020 compared to the pre-COVID-19 period. By 2021, this prevalence had returned to a level similar to before the pandemic. No significant APC changes were observed in the prevalence of mental health.

**Conclusions:**

These findings demonstrate the trends and APCs in health behaviors and mental health conditions among Korean adolescents over the last 5 years. We must pay attention to the heterogeneous and multifaceted features of the COVID-19 pandemic.

## Introduction

Many forms of psychopathology tend to increase in severity and prevalence during adolescence. The coronavirus disease (COVID-19) pandemic-related disruptions are likely to exacerbate developmental vulnerabilities to a wide range of poor mental health and health-risk behaviors (1). The COVID-19 pandemic has caused significant changes to adolescents' daily lives, including stress related to the transition to online learning, isolation from friends, and spending extensive time with their immediate family (2, 3).

The effects of the COVID-19 pandemic on adolescents varied, with some reporting a deterioration in their mental health, whereas others perceived improvement (4, 5). A recent study showed that depression and anxiety symptoms worsened and increased among adolescents during the pandemic (6, 7). Social connectedness and school disruptions are significantly associated with depressive symptoms (8). However, adolescents' daily stress events decreased more during the pandemic (5, 7, 9, 10). A previous study reported that the COVID-19 pandemic increased home- and health-related stress but showed a trend toward lower school-related stress (11). As the COVID-19 pandemic has had unpredictable effects on adolescents, it is crucial to prioritize monitoring and overseeing their mental health during this time.

As of March 2020, in-person education was suspended for over a year because of the COVID-19 pandemic in South Korea (12, 13). Adolescents began to spend most of their time studying and performing daily activities at home. The COVID-19 pandemic has influenced adolescents' educational experiences, social interactions, and health-related lifestyles (8, 14–18). During the pandemic, it was challenging for adolescents to maintain regular mealtimes, and irregular eating habits caused an imbalance in their bodies (19–21). Moreover, lack of activity increased the obesity rate among adolescents during the COVID-19 lockdowns (22). Adolescent health behaviors influence future adult health behaviors and are linked to developing diseases later in life.

Adolescents' specific developmental needs and characteristics can place them at a unique and heightened risk of adverse health outcomes during public health emergencies. Therefore, understanding their behavior and mental health may be critical to their short-term and long-term psychological well-being (23).

The study hypothesized that compared to before the COVID-19 pandemic, the COVID-19 pandemic could increase the risk of poor mental health and unhealthy behaviors among adolescents. The present study aimed to determine whether adolescents' health behaviors and mental health status have changed in South Korea before and during the COVID-19 pandemic.

## Methods

### Data and study population

We analyzed nationally representative data from the 2017-2021 Korea Youth Risk Behavior Web-Based Survey (KYRBWS). KYRBWS is an anonymous, self-administered, structured questionnaire that uses stratified random sampling. The 2017 KYBRWS was conducted from June to August 2017 and involved 62,276 adolescents from 400 middle and 400 high schools. The 2018 and 2019 KYRBWS comprised 60,040 and 57,303 adolescents, respectively, in both years, conducted from June to August. KYRBWS 2020 and 2021 consisted of 54,948 and 54,848 adolescents, respectively, and was conducted from August to November in both years. The participation rates were 95.8%, 95.6%, 95.3%, 94.9%, and 92.9% in 2017, 2018, 2019, 2020, and 2021, respectively (24).

### Health behavior variables

Health behavior variables were assessed using self-reported inadequate physical activity, obesity, alcohol consumption, and smoking experience. Inadequate physical activity was assessed using the question, “In the past seven days, how many days did you perform more than 60 minutes of physical activity (of any type) that would cause you to run out of breath or increase your heart rate?” Possible responses were “none” or “from one to seven days.” In the current study, inadequate physical activity was reclassified into two categories: yes (from one to seven days) and no (25).

Obesity was calculated using the body mass index (BMI), self-reported height, and weight. Obesity was classified according to the criterion of BMI ≥ 25 kg/m^2^ or more. Alcohol consumption and smoking experience were measured using the following questions: “In the past 30 days, have you had more than one glass of alcohol to drink?” and “In the past 30 days, have you smoked at least one cigarette?” The two responses were “yes” or “no” (24).

### Mental health variables

Mental health variables were assessed using self-reported perceived severe stress, depressive mood, and suicide plans and attempts. Extreme perceived stress was measured using the question, “Generally, how do you perceive your stress?” The five responses included “very severe,” “severe,” “moderate,” “little,” and “never.” Our study classified perceived severe stress into two groups: yes (very severe or severe) and no (moderate, little, or never).

Depressive mood was assessed using the following question: “In the past 12 months, have you ever felt sadness or despair that was sufficient to make you pause the daily activities for two whole weeks?” The two responses were “yes” or “no.”

Suicidal ideation was assessed using the question, “In the past 12 months, have you ever seriously considered suicide?” The question assessed suicidal plans: “In the past 12 months, have you ever made any specific plans for suicide?” Suicide attempts were assessed by asking, “In the past 12 months, have you attempted suicide?” The two response options were “yes” or “no” (24).

### Sociodemographic variables

Sociodemographic variables were assessed using sex, type of school, and subjective family income. Subjective family income was assessed using the question: “How would you evaluate your family income?” The five responses included “high,” “middle-high,” “middle,” “low-middle,” and “low.” Subjective family income levels were reclassified into three groups: high, middle (middle-high and middle), and low (low-middle and low) (25).

### Statistical analysis

All statistical analyses were conducted using a multi-stage cluster-sampling design. We stratified health behaviors and mental health effects according to gender. Moreover, subgroup analysis was performed according to the type of school and subjective family income. Prevalence was expressed as a number and weighted percentage to account for nationally representative estimates. The weighted prevalence and 95% confidence intervals (CI) were calculated using SPSS Windows software version 25.0. APC was calculated by inputting the estimates and standard errors from the SPSS into the Joinpoint program. The study was approved by the IRB of the College of Medicine, Catholic University of Korea (IRB approval number: MC22ZISI0048).

## Results

Table 1 shows the sociodemographic characteristics of 289,415 Korean adolescents. In total, 53.5%, 50.4%, and 49.0% were high school students in 2017–2019, 2020, and 2021, respectively. The highest level of subjective family income was 40.3% in 2017–2019, 39.9% in 2020, and 40.1% in 2021. Approximately 50% of respondents answered that their subjective family income was at the middle level for both sexes.

**Table 1:** Sociodemographic characteristics in the KYRBS 2017–2019, 2020, and 2021 samples by sex stratification (*n* = 289,415).

**Characteristics**	**Total**						**Boys**						**Girls**					
	**2017–2019 (***n =*** 179,619)**		**2020 (***n** =* 54,948)**		**2021 (***n** =* 54,848)**		**2017–2019 (***n** =* 91,928)**		**2020 (***n** =* 28,353)**		**2021 (***n** =* 28,401)**		**2017–2019 (***n** =* 87,691)**		**2020 (***n** =* 26,595)**		**2021 (***n** =* 26,447)**	
	**No**.	**Weighted% (95% CI)**	**No**.	**Weighted% (95% CI)**	**No**.	**Weighted% (95% CI)**	**No**.	**Weighted% (95% CI)**	**No**.	**Weighted% (95% CI)**	**No**.	**Weighted% (95% CI)**	**No**.	**Weighted% (95% CI)**	**No**.	**Weighted% (95% CI)**	**No**.	**Weighted% (95% CI)**
**Types of school**																		
Middle school	90,498	46.5 (45.6, 47.3)	28,961	49.6 (48.1, 51.1)	30,015	51.0 (49.5, 52.4)	46,381	46.3 (44.8, 47.9)	14,830	49.4 (46.7, 52.0)	15,586	50.8 (48.2, 53.3)	44,117	46.6 (45.0, 48.3)	14,131	49.9 (47.2, 52.6)	14,429	51.2 (48.4, 53.9)
High school	89,121	53.5 (52.7, 54.4)	25,987	50.4 (48.9, 51.9)	24,833	49.0 (47.6, 50.5)	54,547	53.7 (52.1, 55.2)	13,523	50.6 (48.0, 53.3)	12,815	49.2 (46.7, 51.8)	43,574	53.4 (51.7, 55.0)	12,464	50.1 (47.4, 52.8)	12,018	48.8 (46.1, 51.6)
**Subjective family income**																		
High	71,514	40.3 (39.9, 40.7)	21,339	39.9 (39.1, 40.7)	21,568	40.1 (39.3, 41.0)	39,380	43.1 (42.5, 43.7)	11,623	42.1 (41.1, 43.1)	11,811	42.2 (41.2, 43.2)	32,134	37.3 (36.7, 37.9)	9,716	37.5 (36.5, 38.6)	9,757	37.9 (36.9, 39.0)
Middle	83,847	46.4 (46.1, 46.7)	26,397	47.5 (46.9, 48.2)	27,077	49.0 (48.3, 49.6)	40,610	44.0 (43.6, 44.4)	13,013	45.2 (44.4, 46.0)	13,321	46.6 (45.8, 47.4)	43,237	49.0 (48.6, 49.5)	13,384	50.0 (49.2, 50.8)	13,756	51.5 (50.7, 52.4)
Low	24,258	13.3 (13.0, 13.5)	7,212	12.6 (12.2, 13.0)	6,203	10.9 (10.5, 11.3)	11,938	12.9 (12.6, 13.2)	3,717	12.7 (12.3, 13.2)	3,269	11.2 (10.8, 11.7)	12,320	13.7 (13.3, 14.0)	3,495	12.5 (11.9, 13.0)	2,934	10.5 (10.0, 11.1)

Table 2 shows the weighted prevalence of alcohol consumption and smoking history. The prevalence of alcohol consumption among boys was 44.5 (95% CI, 43.9–45.1) in 2017–2019, 37.5% (95% CI, 36.5–38.5) in 2020, and 37.6% (95% CI, 36.7–38.6) in 2021. The prevalence of alcohol consumption among girls declined during the COVID-19 pandemic compared to the pre-COVID-19 period, except for girls from the low-income level. In contrast, in the annual change in alcohol consumption over the 5 years, both sexes experienced an increase in 2018 compared to 2017 and a decrease again in 2019. In addition, smoking experiences decreased in boys (19.7% in 2017–2019, 13.9% in 2020, and 13.1% in 2021). However, smoking prevalence among girls did not show any similar changes. Girls from middle- and low-income levels were more likely to have had smoking experience in 2020 and 2021 than in 2017–2019.

**Table 2:** Weighted prevalence of alcohol consumption and smoking experience in the KYRBS 2017–2019, 2020, and 2021 samples^*^.

**Characteristics**	**Alcohol consumption**			**Smoking experience**		
	**2017–2019 (*****n** =* **71,354)**	**2020 (*****n** =* **18,357)**	**2021 (*****n** =* **17,939)**	**2017–2019 (*****n** =* **23,766)**	**2020 (*****n** =* **5,630)**	**2021 (*****n** =* **5,329)**
**Total (weighted %)**	40.6 (40.2, 41.1)	33.4 (32.7, 34.2)	32.9 (32.2, 33.6)	13.8 (13.5, 14.2)	10.2 (9.7, 10.7)	9.9 (9.5, 10.4)
**Types of school**						
Middle school	27.0 (26.6, 27.4)	21.8 (21.2, 22.5)	22.2 (21.5, 22.9)	7.8 (7.5, 8.0)	5.1 (4.8, 5.5)	4.7 (4.4, 5.0)
High school	52.5 (51.9, 53.1)	44.9 (43.9, 45.9)	44.0 (43.0, 45.1)	19.1 (18.5, 19.7)	15.2 (14.4, 16.1)	15.3 (14.5, 16.1)
**Subjective family income**						
High	37.4 (36.9, 38.0)	30.1 (29.2, 31.0)	30.0 (29.1, 30.9)	12.6 (12.2, 13.0)	9.4 (8.9, 10.0)	9.0 (8.5, 9.5)
Middle	40.7 (40.2, 41.2)	33.5 (32.7, 34.4)	33.0 (32.1, 33.8)	13.1 (12.7, 13.5)	9.6 (9.1, 10.1)	9.4 (9.0, 9.9)
Low	50.1 (49.4, 50.8)	43.7 (42.4, 45.0)	43.2 (41.8, 44.5)	19.9 (19.2, 20.5)	15.1 (14.0, 16.2)	15.3 (14.3, 16.4)
**Boys (weighted %)**	44.5 (43.9, 45.1)	37.5 (36.5, 38.5)	37.6 (36.7, 38.6)	19.7 (19.2, 20.1)	13.9 (13.2, 14.6)	13.1 (12.5, 13.8)
**Types of school**						
Middle school	30.5 (30.0, 31.1)	25.1 (24.2, 25.9)	26.2 (25.3, 27.1)	10.7 (10.3, 11.1)	6.5 (6.0, 6.9)	5.8 (5.4, 6.2)
High school	56.5 (55.9, 57.2)	49.6 (48.3, 50.8)	49.4 (48.3, 50.6)	27.4 (26.7, 28.0)	21.1 (19.9, 22.2)	20.7 (19.7, 21.8)
**Subjective family income**						
High	41.5 (40.8, 42.2)	34.2 (32.9, 35.5)	35.0 (33.8, 36.2)	17.7 (17.2, 18.3)	12.5 (11.7, 13.4)	12.2 (11.4, 13.0)
Middle	45.1 (44.4, 45.8)	37.8 (36.7, 38.9)	38.0 (35.9, 39.1)	19.5 (19.0, 20.1)	13.3 (12.5, 14.1)	12.7 (12.0, 13.5)
Low	52.3 (51.3, 53.3)	47.2 (45.4, 49.1)	46.0 (44.2, 47.9)	26.5 (25.6, 27.4)	20.1 (18.6, 21.7)	18.6 (17.2, 20.1)
**Girls (weighted %)**	36.5 (35.8, 37.1)	29.1 (28.1, 30.1)	27.8 (26.9, 28.8)	7.5 (7.2, 7.8)	6.3 (5.9, 6.7)	6.4 (6.0, 6.8)
**Types of school**						
Middle school	23.2 (22.6, 23.7)	18.4 (17.5, 19.3)	17.9 (17.1, 18.8)	4.6 (4.3, 4.8)	3.7 (3.4, 4.1)	3.6 (3.3, 4.0)
High school	48.1 (47.2, 48.9)	39.8 (38.4, 41.1)	38.2 (36.9, 39.6)	10.0 (9.5, 10.5)	8.8 (8.1, 9.5)	9.4 (8.7, 10.2)
**Subjective family income**						
High	32.3 (31.5, 33.1)	25.1 (24.0, 26.3)	24.2 (23.0, 25.3)	6.2 (5.9, 6.5)	5.6 (5.1, 6.2)	5.2 (4.7, 5.7)
Middle	36.4 (35.7, 37.1)	29.4 (28.3, 30.6)	28.1 (27.1, 29.1)	6.9 (6.5, 7.2)	5.9 (5.5, 6.4)	6.3 (5.8, 6.8)
Low	47.9 (46.9, 48.9)	39.8 (38.0, 41.6)	39.9 (38.1, 41.8)	13.0 (12.3, 13.8)	9.5 (8.4, 10.8)	11.6 (10.4, 13.0)

^*^Values are presented as weighted percentages (95% confidence intervals).

Table 3 lists the weighted prevalence of inadequate physical activity and obesity. The prevalence of inadequate physical activity for both sexes increased in 2020 compared to the pre-COVID-19 period and decreased again in 2021 (boys: 26.9% in 2017–2019, 30.2% in 2020, and 26.1% in 2021; girls: 46.0% in 2017–2019, 48.7% in 2020, and 42.5% in 2021). For both, the prevalence of obesity increased in 2020–2021 compared to 2017–2019 (boys, 19.8% in 2017–2019, 23.3% in 2020, and 25.4% in 2021; girls, 9.2% in 2017–019, 9.7% in 2020, and 10.3% in 2021). From 2017 to 2021, the prevalence of obesity among boys was more than twice that of girls. Furthermore, its prevalence in girls from the low-income level (15.3, 95% CI 13.9–16.8) was approximately twice that of girls from the high-income level (8.7, 95% CI 8.1–9.2) in 2021.

**Table 3:** The weighted prevalence of inadequate physical activity and obesity in the KYRBS 2017-2019, 2020, and 2021 samples.

**Characteristics**	**Inadequate physical activity**			**Obesity**		
	**2017-2019 (*****n** =* **63,938)**	**2020 (*****n** =* **21,111)**	**2021 (*****n** =* **18,250)**	**2017-2019 (*****n** =* **25,469)**	**2020 (*****n** =* **9,093)**	**2021 (*****n** =* **9,738)**
**Total (weighted %)**	36.0 (35.6, 36.5)	39.1 (38.4, 39.8)	34.0 (33.4, 34.7)	14.7 (14.4, 15.0)	16.8 (16.2, 17.3)	18.1 (17.6, 18.7)
**Types of school**						
Middle school	33.4 (33.0, 33.9)	35.4 (34.6, 36.2)	29.6 (28.9, 30.3)	11.1 (10.8, 11.4)	13.9 (13.3, 14.6)	15.3 (14.7, 15.9)
High school	38.3 (37.6, 39.0)	42.8 (41.6, 44.0)	38.7 (37.5, 39.8)	17.8 (17.4, 18.2)	19.6 (18.8, 20.4)	21.1 (20.2, 21.9)
**Subjective family income**						
High	33.9 (33.4, 34.4)	35.4 (34.6, 36.3)	31.2 (30.4, 32.0)	14.0 (13.6, 14.3)	15.9 (15.3, 16.6)	17.3 (16.7, 18.0)
Middle	37.2 (36.7, 37.8)	41.4 (40.5, 42.2)	35.7 (34.9, 36.5)	14.2 (13.9, 14.5)	16.2 (15.6, 16.9)	18.0 (17.3, 18.6)
Low	38.3 (37.5, 39.0)	42.5 (41.1, 43.9)	36.9 (35.6, 38.3)	18.8 (18.2, 19.3)	21.4 (20.3, 22.5)	21.7 (20.6, 22.8)
**Boys (weighted %)**	26.9 (26.5, 27.3)	30.2 (29.5, 30.9)	26.1 (25.5, 26.7)	19.8 (19.4, 20.1)	23.3 (22.7, 23.9)	25.4 (24.7, 26.0)
**Types of school**						
Middle school	26.9 (26.5, 27.4)	28.9 (28.1, 29.7)	23.9 (23.1, 24.7)	15.6 (15.2, 16.0)	20.4 (19.6, 21.3)	22.5 (21.8, 23.3)
High school	26.8 (26.2, 27.4)	31.6 (30.5, 32.7)	28.3 (27.4, 29.3)	23.4 (23.0, 23.9)	26.2 (25.3, 27.0)	28.4 (27.4, 29.3)
**Subjective family income**						
High	25.8 (25.3, 26.3)	27.7 (26.8, 28.7)	24.1 (23.3, 25.0)	19.0 (18.5, 19.4)	22.6 (21.7, 23.5)	24.6 (23.7, 25.5)
Middle	27.3 (26.8, 27.8)	31.7 (30.8, 32.6)	27.0 (26.2, 27.8)	19.5 (19.1, 19.9)	23.0 (22.1, 23.8)	25.7 (24.8, 26.5)
Low	29.1 (28.2, 30.0)	33.4 (31.8, 35.0)	29.8 (28.1, 31.5)	23.4 (22.6, 24.2)	27.0 (25.5, 28.5)	27.2 (25.7, 28.9)
**Girls (weighted %)**	46.0 (45.5, 46.5)	48.7 (47.9, 49.6)	42.5 (41.7, 43.4)	9.2 (9.0, 9.5)	9.7 (9.2, 10.1)	10.3 (9.9, 10.7)
**Types of school**						
Middle school	40.4 (39.8, 41.0)	42.4 (41.3, 43.5)	35.6 (34.7, 36.5)	6.3 (6.1, 6.6)	7.0 (6.5, 7.5)	7.6 (7.1, 8.1)
High school	50.9 (50.2, 51.5)	55.0 (53.9, 56.1)	49.8 (48.6, 51.0)	11.7 (11.4, 12.1)	12.4 (11.7, 13.0)	13.1 (12.5, 13.8)
**Subjective family income**						
High	44.2 (43.5, 44.8)	44.7 (43.5, 45.9)	39.7 (38.5, 40.9)	7.7 (7.4, 8.1)	7.9 (7.3, 8.5)	8.7 (8.1, 9.2)
Middle	46.9 (46.4, 47.5)	50.8 (49.8, 51.8)	44.2 (43.1, 45.2)	9.0 (8.7, 9.3)	9.7 (9.1, 10.3)	10.5 (9.9, 11.1)
Low	47.7 (46.7, 48.6)	52.5 (50.7, 54.4)	45.1 (43.1, 47.1)	14.0 (13.4, 14.7)	15.2 (13.9, 16.5)	15.3 (13.9, 16.8)

^*^Values are presented as weighted percentages (95% confidence intervals).

**Figure 1:**
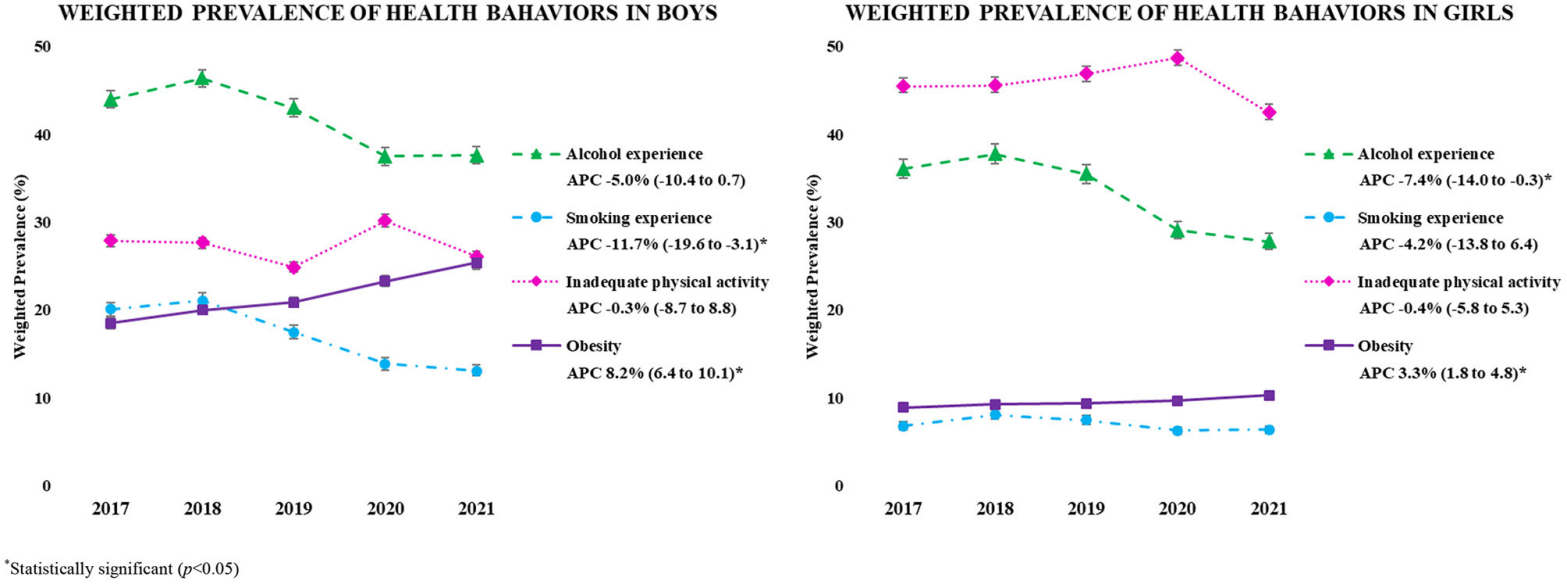
The weighted prevalence of health behavior in the KYRBS 2017-2021 samples. ^*^Statistically significant (*p* < 0.05).

Figure 1 shows the recent trends and APC (2017–2021) in health behaviors (alcohol/smoking experience, inadequate physical activity, and obesity) of Korean adolescents by sex. The prevalence of obesity in both sexes increased regardless of the period (boys, APC = 8.2%, 95% CI, 6.4–10.1; girls, APC = 3.3%, 95% CI, 1.8–4.8). Smoking experience decreased for boys (APC = −11.7%, 95% CI, −19.6 to −3.1), and alcohol consumption decreased for girls (APC = −7.4%, 95% CI, −14.0 to −0.3).

Table 4 shows the weighted prevalence of perceived severe stress and depressive moods. The prevalence of stress and depression was higher in girls than in boys over the 5 years. In particular, boys' perceived severe stress decreased in 2020 compared to 2017–2019. However, it increased in 2021 compared with the pre-COVID-19 period (31.4% in 2017–2019, 28.1% in 2020, and 32.3% in 2021). The prevalence of perceived severe stress among girls was 47.6 (95% CI, 47.1–48.0) in 2017–2019, which decreased to 40.7% (95% CI, 39.9–41.4) in 2020 and increased to 45.6% (95% CI, 44.9–46.3) in 2021. Additionally, increased depressive mood patterns in both sexes were noticeable in the second year of the COVID-19 pandemic, similar to the level before COVID-19. The prevalence of depressive mood among boys and girls from the low-income level was the highest in 2021 compared to the pre-COVID-19 pandemic period (boys: 29.7% in 2017–2019, 28.7% in 2020, and 30.9% in 2021; girls: 43.7% in 2017–2019, 41.3% in 2020, and 43.8% in 2021).

**Table 4:** The weighted prevalence of perceived severe stress and depressive mood in the KYRBS 2017–2019, 2020, and 2021 samples^*^.

**Characteristics**	**Perceived severe stress**			**Depressive mood**		
	**2017-2019 (*****n** =* **70,349)**	**2020 (*****n** =* **18,662)**	**2021 (*****n** =* **21,245)**	**2017-2019 (*****n** =* **47,848)**	**2020 (*****n** =* **13,840)**	**2021 (*****n** =* **14,692)**
**Total (weighted %)**	39.1 (38.6, 39.6)	34.2 (33.4, 34.9)	38.7 (38.0, 39.6)	26.7 (26.4, 27.0)	25.2 (24.7, 25.7)	26.8 (26.3, 27.3)
**Types of school**						
Middle school	36.1 (35.6, 36.5)	30.4 (29.7, 31.1)	36.4 (35.7, 37.1)	25.1 (24.8, 25.5)	22.9 (22.3, 23.6)	25.9 (25.3, 26.6)
High school	41.8 (41.2, 42.3)	37.9 (37.0, 38.7)	41.2 (40.3, 42.1)	28.1 (27.7, 28.5)	27.4 (26.6, 28.2)	27.7 (26.9, 28.4)
**Subjective family income**						
High	35.5 (35.1, 36.0)	30.8 (30.0, 31.5)	36.6 (35.9, 37.4)	24.9 (24.5, 25.3)	23.3 (22.6, 23.9)	25.4 (24.8, 26.0)
Middle	38.5 (38.1, 39.0)	33.6 (32.9, 34.3)	37.8 (37.0, 38.5)	25.5 (25.1, 25.9)	24.3 (23.7, 24.9)	25.6 (25.0, 26.3)
Low	52.1 (51.4, 52.8)	47.1 (45.8, 48.3)	50.9 (49.6, 52.2)	36.6 (35.9, 37.2)	34.7 (33.6, 35.9)	36.9 (35.6, 38.2)
**Boys (weighted %)**	31.4 (31.0, 31.7)	28.1 (27.4, 28.8)	32.3 (31.7, 32.9)	21.2 (20.8, 21.5)	20.1 (19.5, 20.7)	22.4 (21.9, 23.0)
**Types of school**						
Middle school	28.9 (28.5, 29.4)	24.9 (24.2, 25.7)	31.5 (30.6, 32.4)	19.2 (18.8, 19.6)	17.8 (17.1, 18.6)	21.7 (21.0, 22.5)
High school	33.5 (32.9, 34.0)	31.1 (30.2, 32.1)	33.2 (32.3, 34.0)	22.8 (22.4, 23.3)	22.2 (21.4, 23.1)	23.1 (22.3, 24.0)
**Subjective family income**						
High	28.7 (28.2, 29.2)	25.6 (24.8, 26.5)	31.3 (30.4, 32.3)	20.1 (19.7, 20.5)	18.7 (18.0, 19.5)	22.1 (21.3, 22.9)
Middle	30.3 (29.8, 30.8)	27.1 (26.3, 28.0)	30.3 (29.5, 31.2)	19.7 (19.3, 20.1)	18.9 (18.1, 19.7)	20.7 (19.9, 21.5)
Low	43.8 (42.9, 44.7)	39.6 (38.0, 41.3)	44.3 (42.6, 46.1)	29.7 (28.8, 30.5)	28.7 (27.2, 30.3)	30.9 (29.2, 32.6)
**Girls (weighted %)**	47.6 (47.1, 48.0)	40.7 (39.9, 41.4)	45.6 (44.9, 46.3)	32.8 (32.4, 33.1)	30.7 (30.0, 31.4)	31.4 (30.8, 32.1)
**Types of school**						
Middle school	43.8 (43.2, 44.4)	36.2 (35.3, 37.1)	41.5 (40.6, 42.5)	31.6 (31.0, 32.1)	28.4 (27.5, 29.2)	30.4 (29.6, 31.2)
High school	50.9 (50.3, 51.5)	45.2 (44.1, 46.2)	49.9 (49.0, 50.9)	33.8 (33.3, 34.4)	33.0 (32.0, 34.0)	32.5 (31.6, 33.5)
**Subjective family income**						
High	44.1 (43.5, 44.8)	37.0 (35.9, 38.1)	43.0 (41.9, 44.0)	30.9 (30.3, 31.4)	28.7 (27.8, 29.6)	29.4 (28.5, 30.3)
Middle	46.5 (46.0, 47.1)	39.8 (38.9, 40.8)	45.0 (44.1, 45.9)	31.1 (30.6, 31.7)	29.6 (28.7, 30.4)	30.4 (29.6, 31.3)
Low	60.6 (59.8, 61.5)	55.3 (53.6, 56.9)	58.4 (56.7, 60.1)	43.7 (42.8, 44.5)	41.3 (39.7, 43.0)	43.8 (41.9, 45.7)

^*^Values are presented as weighted percentages (95% confidence intervals).

Table 5 shows the weighted prevalence of suicidal behaviors. For both boys and girls, the prevalence of suicidal ideation, plans, and attempts decreased in 2020 compared to pre-COVID-19 and increased in 2021. In particular, suicidal ideation and planning among girls from a low-income level showed the highest prevalence in the second year of the COVID-19 pandemic compared to pre-COVID-19. For example, 28.0% (95% CI 26.3–29.7) of girls from the low-income level reported suicidal ideation and plan in 2021 compared to 26.0% (95% CI 25.1–26.8) in 2017–2019.

**Table 5:** The weighted prevalence of suicidal behavior in the KYRBS 2017-2019, 2020, and 2021 samples^*^.

**Characteristics**	**Suicidal ideation**			**Suicidal plan**			**Suicidal attempt**		
	**2017-2019 (*****n** =* **23,058)**	**2020 (*****n** =* **5,979)**	**2021 (*****n** =* **6,956)**	**2017-2019 (*****n** =* **7,418)**	**2020(*****n** =* **1,953)**	**2021(*****n** =* **2,206)**	**2017-2019 (*****n** =* **5,238)**	**2020(*****n** =* **1,121)**	**2021(*****n** =* **1,245)**
**Total (weighted %)**	12.8 (12.6, 13.0)	10.9 (10.5, 11.2)	12.7 (12.4, 13.1)	4.1 (4.0, 4.2)	3.6 (3.4, 3.7)	4.0 (3.8, 4.2)	2.9 (2.9, 3.0)	2.0 (1.9, 2.1)	2.2 (2.1, 2.3)
**Types of school**						
Middle school	13.5 (13.2, 13.8)	10.2 (9.8, 10.7)	13.4 (12.9, 13.9)	4.7 (4.6, 4.9)	3.7 (3.4, 3.9)	4.4 (4.1, 4.7)	3.4 (3.3, 3.5)	2.0 (1.8, 2.2)	2.4 (2.2, 2.6)
High school	12.2 (11.9, 12.5)	11.5 (11.0, 12.0)	12.0 (11.5, 12.6)	3.6 (3.4, 3.7)	3.4 (3.2, 3.7)	3.6 (3.4, 3.9)	2.4 (2.3, 2.5)	2.0 (1.9, 2.2)	2.0 (1.9, 2.2)
**Subjective family income**						
High	11.4 (11.1, 11.6)	9.5 (9.1, 10.0)	11.3 (10.9, 11.8)	3.8 (3.7, 3.9)	3.2 (3.0, 3.5)	3.7 (3.4, 4.0)	2.6 (2.4, 2.7)	1.8 (1.6, 2.0)	1.9 (1.7, 2.1)
Middle	11.8 (11.5, 12.0)	10.1 (9.7, 10.5)	11.9 (11.4, 12.4)	3.4 (3.3, 3.5)	3.1 (2.9, 3.3)	3.4 (3.2, 3.7)	2.4 (2.3, 2.5)	1.7 (1.5, 1.9)	1.9 (1.7, 2.0)
Low	20.9 (20.4, 21.4)	18.2 (17.3, 19.2)	21.6 (20.5, 22.7)	7.5 (7.1, 7.8)	6.4 (5.8, 7.0)	8.0 (7.2, 8.8)	5.5 (5.2, 5.8)	3.9 (3.5, 4.4)	4.8 (4.3, 5.3)
**Boys (weighted %)**	9.5 (9.2, 9.7)	8.1 (7.7, 8.5)	9.5 (9.1, 9.9)	3.3 (3.2, 3.4)	2.8 (2.6, 3.0)	3.2 (3.0, 3.4)	2.0 (1.9, 2.1)	1.4 (1.2, 1.5)	1.5 (1.4, 1.7)
**Types of school**						
Middle school	9.4 (9.1, 9.7)	7.4 (7.0, 7.9)	10.0 (9.5, 10.6)	3.6 (3.4, 3.7)	2.8 (2.5, 3.1)	3.4 (3.1, 3.8)	2.2 (2.1, 2.4)	1.3 (1.1, 1.5)	1.6 (1.4, 1.9)
High school	9.6 (9.2, 9.9)	8.8 (8.2, 9.3)	9.0 (8.5, 9.6)	3.1 (2.9, 3.3)	2.7 (2.5, 3.1)	2.9 (2.6, 3.3)	1.9 (1.7, 2.0)	1.4 (1.2, 1.6)	1.4 (1.2, 1.6)
**Subjective family income**						
High	8.6 (8.3, 9.0)	7.1 (6.6, 7.6)	8.9 (8.4, 9.5)	3.3 (3.1, 3.5)	2.7 (2.4, 3.0)	3.1 (2.8, 3.4)	2.1 (1.9, 2.2)	1.3 (1.1, 1.6)	1.4 (1.2, 1.6)
Middle	8.4 (8.1, 8.6)	7.5 (7.0, 8.0)	8.5 (8.0, 9.1)	2.6 (2.4, 2.7)	2.2 (2.0, 2.5)	2.7 (2.4, 3.0)	1.5 (1.4, 1.6)	1.0 (0.9, 1.2)	1.2 (1.1, 1.5)
Low	16.0 (15.3, 16.7)	13.5 (12.5, 14.7)	15.9 (14.7, 17.2)	6.0 (5.5, 6.4)	5.0 (4.3, 5.7)	6.0 (5.1, 7.0)	3.8 (3.5, 4.2)	2.5 (2.0, 3.0)	3.2 (2.7, 3.9)
**Girls (weighted %)**	16.5 (16.2, 16.8)	13.9 (13.4, 14.4)	16.1 (15.6, 16.6)	4.9 (4.8, 5.1)	4.4 (4.1, 4.7)	4.9 (4.6, 5.2)	3.8 (3.6, 3.9)	2.7 (2.6, 3.0)	2.9 (2.7, 3.2)
**Types of school**						
Middle school	18.0 (17.6, 18.4)	13.3 (12.6, 14.0)	16.9 (16.2, 17.6)	6.0 (5.7, 6.2)	4.6 (4.2, 5.0)	5.4 (5.0, 5.9)	4.7 (4.5, 4.9)	2.8 (2.5, 3.1)	3.1 (2.8, 3.5)
High school	15.1 (14.7, 15.5)	14.5 (13.8, 15.2)	15.3 (14.6, 16.1)	4.0 (3.8, 4.3)	4.2 (3.9, 4.6)	4.4 (4.0, 4.8)	3.0 (2.8, 3.2)	2.7 (2.5, 3.0)	2.7 (2.5, 3.0)
**Subjective family income**						
High	14.8 (14.4, 15.2)	12.4 (11.7, 13.2)	14.1 (13.4, 14.9)	4.4 (4.2, 4.7)	3.9 (3.5, 4.3)	4.4 (4.0, 4.9)	3.2 (3.0, 3.4)	2.4 (2.1, 2.7)	2.6 (2.3, 2.9)
Middle	15.1 (14.7, 15.5)	12.6 (12.0, 13.3)	15.1 (14.4, 15.9)	4.2 (4.0, 4.4)	3.9 (3.6, 4.3)	4.2 (3.8, 4.6)	3.3 (3.1, 3.4)	2.3 (2.1, 2.6)	2.5 (2.2, 2.7)
Low	26.0 (25.1, 26.8)	23.4 (21.9, 24.9)	28.0 (26.3, 29.7)	9.0 (8.5, 9.5)	7.9 (7.0, 8.9)	10.3 (9.2, 11.5)	7.2 (6.7, 7.6)	5.6 (4.9, 6.4)	6.5 (5.7, 7.5)

^*^Values are presented as weighted percentages (95% confidence intervals).

**Figure 2:**
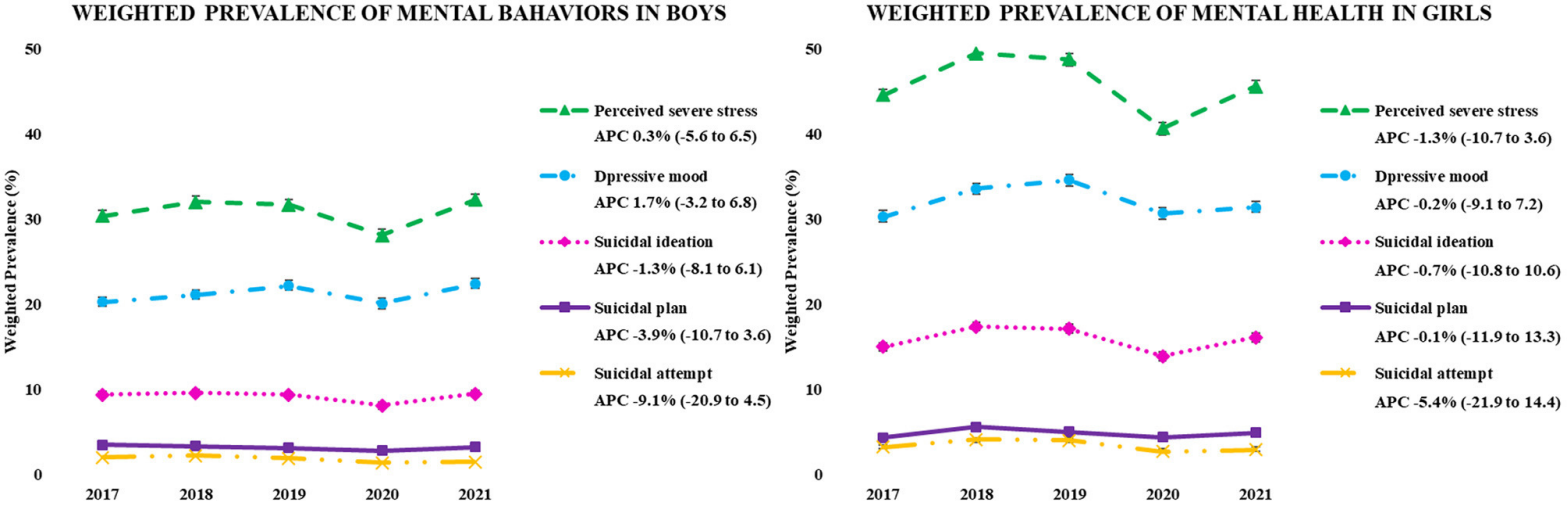
The weighted prevalence of mental health in the KYRBS 2017–2021 samples.

Figure 2 depicts the recent trends and APC (2017–2021) in the weighted prevalence of mental health (perceived severe stress, depressive mood, and suicidal behavior) of Korean adolescents by gender. No significant APCs changes were observed in the prevalence of mental health.

## Discussion

This study used nationally representative data to present 5-year trends from 2017 to 2021 for health behaviors and mental health in the Korean adolescent population. The present study found that the prevalence of drinking among boys and girls decreased in the first and second years of the COVID-19 pandemic compared to the pre-COVID-19 period. Smoking experiences decreased among boys. After social distancing began, significantly fewer teens reported binge drinking or vaping (26, 27). Social distancing limited adolescents' interactions with peers and led to decreased smoking and alcohol consumption during the COVID-19 pandemic (28–30). It was more challenging to access substances and circumvent parental supervision during the 3–6 weeks after the initial stay-at-home orders (30). In addition, remote learning does not require early morning start times, thus alleviating sleep deprivation, which is a risk factor for substance use (30). Stay-at-home and social distancing orders might create unexpected benefits in reducing adolescents' substance use.

This study showed that inadequate physical activity among the sexes increased in 2020 and decreased again in 2021. The physical activity level adopted during the social distancing period was significantly lower than that before the COVID-19 pandemic (26, 31). Despite positive changes in physical activity in 2021 compared with 2020, the prevalence of obesity continuously increased in 2021 compared to 2020 for boys and girls.

The present study demonstrated that the mental health of both boys and girls improved in the first year of the COVID-19 pandemic compared with the pre-COVID-19 pandemic period. Our results were consistent with those of a previous study showing that adolescents' mental health improved in the first year of the pandemic (32). It could be speculated that school closures temporarily relieved adolescents who experienced pressure and burden due to school work (33). Remote or hybrid learning offers less anxiety and stress for teens who experience academic or social pressure at school (30). This decreased stress may have decreased substance use by teens who consume alcohol and smoke to cope with stress and its adverse effects.

Notably, our findings showed that an increase in depressive mood and perceived severe stress among boys and girls was noticeable during the second year of the COVID-19 pandemic. Furthermore, the prevalence of suicidal ideation, plans, and attempts for both boys and girls increased in 2021 compared to 2020. These findings support previous studies showing that the COVID-19 outbreak had a detrimental impact on adolescent mental health (4, 17, 22). The Korean government policy combined offline and online classes by adjusting the number of students who attended school to two-thirds in 2021 (21, 34). In the second year of the pandemic, students were required to adjust to the evolving educational policies and quarantine guidelines. Consequently, they expressed their challenges with increased levels of confusion and stress (20, 27). Moreover, adolescents may feel overwhelmed by prolonged media coverage (35). As the duration of the pandemic increases, it may be beneficial to provide vulnerable adolescents with access to mental health services and social resources to help manage their stress and depressive symptoms.

The current findings showed that the prevalence of depressive mood and suicidal ideation among boys and girls from low-income levels was the highest over 5 years (2017–2021). Specifically, in the second year of the COVID-19 outbreak, it was found that adolescents from low-income levels had higher rates of maladaptive health behaviors and mental health problems than those from high-income levels. This suggests that adolescent girls from low-income families are especially vulnerable to the effects of COVID-19, which can affect their mental health. Girls from disadvantaged backgrounds face the most significant mental health risks triggered by the COVID-19 pandemic (36). Interventions seeking to mitigate mental health impacts on this vulnerable population must respond to the unpredictable pandemic environment (37).

In summary, smoking and alcohol consumption decreased, and mental health improved in the first year of the COVID-19 pandemic compared with before the pandemic. However, in 2021, the second year of the pandemic, these trends have returned to similar levels. The uncertainty or anxiety symptoms associated with the COVID-19 pandemic may have contributed to increased stress levels, which could lead to unhealthy behaviors and suicide risk (38). However, because each country responded differently to the COVID-19 pandemic and lockdown of schools, it is necessary to compare the results with those from previous studies (39, 40). Additionally, the heterogeneous and multifaceted characteristics of the protracted COVID-19 pandemic should be considered.

This study had some limitations. First, we used secondary data from a national sample. Therefore, it was impossible to infer a causal relationship based on the cross-sectional characteristics of the data. Second, a single question was used for the measurement of mental health. A clinical diagnosis of stress or depressive mood by a clinician was not obtained. Standardized scales for suicidal ideation or attempts were not used. Therefore, our results have limited interpretability with respect to the changes in the severity and prevalence of mental health. Third, participants were asked to provide subjective perceptions of family income instead of using absolute socioeconomic status measures. Future research that includes parents' educational attainment, neighborhood deprivation, or objective income is needed. Fourth, adolescents may have overreported or reported socially acceptable personality characteristics, rather than their true selves. Fifth, because the results are based on secondary data, we did not consider any COVID-19 measures, such as pandemic severity or level of lockdown. Despite these limitations, our findings identified trends in adolescents' health behaviors and mental health conditions while considering the changes over 5 years using national data.

In conclusion, these results indicate the importance of recent trends and APC in adolescent health behaviors and mental health conditions in Korea over 5 years (2017–2021) and focusing on the COVID-19 outbreak. Our results showed that perceived severe stress, depression, suicidal ideation, planning, and attempts temporarily decreased in the first year of the pandemic and increased again in the second year. As the COVID-19 pandemic persists in Korea and schools alternate between face-to-face and online instruction, adolescents may face increased mental health issues as they return to school (32). Therefore, school policymakers and parents should pay special attention to the mental health needs of students during the readjustment period. Additionally, the impact of the unusual pandemic on adolescents should be considered by implementing assessment and early intervention to reduce adverse damage. These findings could be used as primary data for developing coping strategies that national and school settings may adopt for the next outbreak.

## Data availability statement

The datasets presented in this study can be found in online repositories. The data can be publicly downloaded at http://www.kdca.go.kr/yhs/ after entering basic personal details. We used the SPSS dataset of KYRBS in the year 2017–2021. The authors do not possess the right to directly distribute the data.

## Ethics statement

The study was approved by the Institutional Review Board (IRB) of the College of Medicine, The Catholic University of Korea (IRB approval number: MC22ZISI0048). Written informed consent from the participants' legal guardian/next of kin was not required to participate in this study in accordance with the national legislation and the institutional requirements.

## Author contributions

Conceptualization and validation: M-SL and HL. Methodology, software, writing—review and editing, and visualization: M-SL, DK, and HL. Formal analysis, data curation, and writing—original draft preparation: M-SL. Investigation, supervision, project administration, and funding acquisition: HL. All authors have read and agreed to the published version of the manuscript.
